# Research on the Relationship between Reaction Ability and Mental State for Online Assessment of Driving Fatigue

**DOI:** 10.3390/ijerph13121174

**Published:** 2016-11-24

**Authors:** Mengzhu Guo, Shiwu Li, Linhong Wang, Meng Chai, Facheng Chen, Yunong Wei

**Affiliations:** School of Transportation, Jilin University, No. 5988, Renmin Street, Nanguan District, Changchun 130022, China; ccgmz0304@163.com (M.G.); lshiwu@163.com (S.L.); chaimeng16@126.com (M.C.); chenfc15@mails.jlu.edu.cn (F.C.); weiyn15@mails.jlu.edu.cn (Y.W.)

**Keywords:** traffic safety, mental fatigue, reaction time, physiological signals, gray correlation analysis, support vector machine, genetic algorithm

## Abstract

*Background:* Driving fatigue affects the reaction ability of a driver. The aim of this research is to analyze the relationship between driving fatigue, physiological signals and driver’s reaction time. *Methods:* Twenty subjects were tested during driving. Data pertaining to reaction time and physiological signals including electroencephalograph (EEG) were collected from twenty simulation experiments. Grey correlation analysis was used to select the input variable of the classification model. A support vector machine was used to divide the mental state into three levels. The penalty factor for the model was optimized using a genetic algorithm. *Results:* The results show that α/β has the greatest correlation to reaction time. The classification results show an accuracy of 86%, a sensitivity of 87.5% and a specificity of 85.53%. The average increase of reaction time is 16.72% from alert state to fatigued state. Females have a faster decrease in reaction ability than males as driving fatigue accumulates. Elderly drivers have longer reaction times than the young. *Conclusions:* A grey correlation analysis can be used to improve the classification accuracy of the support vector machine (SVM) model. This paper provides basic research that online detection of fatigue can be performed using only a simple device, which is more comfortable for users.

## 1. Introduction

Driving fatigue is a common phenomenon during driving, and is a hot research topic in the field of traffic safety. Fatigue has a remarkable impact on a driver’s perceptions, attention, decision-making and judgement. The control ability of a vehicle is directly determined by a driver’s reaction ability during driving performance. Statistical analysis of braking reaction time has shown that the average reaction time for drivers without accidents was 0.377 s, and the average reaction time for drivers who had accidents was 0.393 s [1]. The most directly-observable behavior of a driver is a slow response when under fatigue. Hence, research into a driver’s reaction ability is important in the assessment of driving fatigue.

In previous research on driver’s reaction times, the relationship between reaction time and distractions, age, gender, vehicle transmission type, emergency situation, visual task load, stimulus location and different stimuli was studied [2,3,4]. The fact that a driver’s reaction time has a direct effect on the acceleration control pedal, the service brake and the steering wheel has also been previously analyzed [5]. The effects of age and mental workload on the reaction time have been identified [6]. A mathematical formula to estimate a driver’s reaction time in various situations has been developed [7]. Some researchers investigating driving fatigue have chosen a single parameter (electrocardiograph (ECG) or electroencephalograph (EEG)) for driving fatigue assessment [8,9,10,11]. The EEG signal has been recorded and a self-assessment was combined with reaction time to assess driving fatigue. It was found that obvious changes were only seen in terms of the α/β parameter after 60 min of driving [12]. To classify driving fatigue, the EEG signal has been previously chosen as the input variable of classification models based on machine learning [13,14,15]. The ECG and the EEG have also been combined to provide the input variables for classification, and the hidden Markov model, Bayesian networks and the support vector machine (SVM) were chosen for fatigue detection and classification [16,17]. Previous research into the relationship between reaction ability, physiological signals and driving fatigue is not common, as most studies chose physiological signals to assess driving fatigue. Additionally, the measurement devices required for physiological signals are usually large, complex, hard to place onboard and cause interference to the driver. A driver’s reaction ability is closely related to physiological signals, since physiological signals will show a variation if the driver’s reaction ability decreases. A decrease in reaction ability and the ability to control the vehicle may affect driving safety when the driver is fatigued.

In this research, physiological signals (EEG and ECG) and the driver’s reaction time during driving were collected from twenty experiments. The physiological impacting factor with the largest correlation to the reaction time was found by grey correlation analysis. An SVM classification model was established based on the Genetic Algorithm (GA), which was optimized to divide the mental state into three levels. The aim of this research is to study the relationship between a driver’s reaction ability, physiological signals and driving fatigue, in order to analyze the variation in reaction time under different mental states, ages and genders. Moreover, a new method for detection of online driving fatigue is proposed in this paper to reduce the size of the measurement device.

## 2. Methodology

### 2.1. Experiment Design

#### 2.1.1. Experimental Equipment

The experimental equipment includes a simulator, a biopac system, a reaction time test system and a computer. A simulator was used to provide the driving environment. A biopac system was connected to the hardware in order to measure, record and save the physiological signals (EEG, electrooculogram (EOG) and ECG) during the experiment and to analyze the data afterwards.

**Figure 1:**
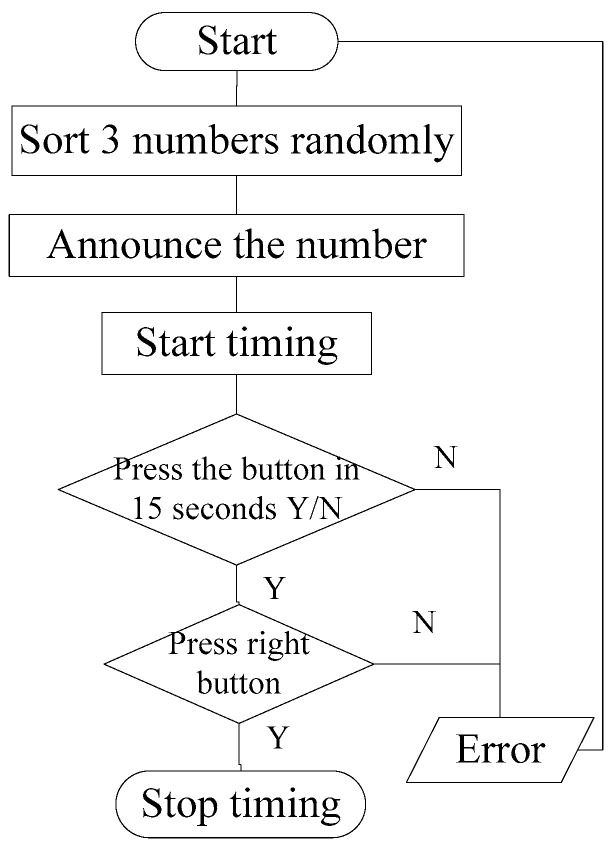
The flow chart of the reaction time test.

The length of time of a reaction determines whether or not the response is timely. Hence, the reaction time was chosen as the measurable characteristic that was used to reflect the reaction ability. The reaction time was measured using a small keyboard affixed to the right side of the steering wheel. It was important to design the reaction time test method independently, and ensure that it would have no effect on driving. Simple reaction time and choice reaction time are the two types of driver’s reaction times. The simple reaction time is the length of time that a driver spends making a single response to a single stimulus signal. However, there is a lot of information that a driver needs to consider to make decisions during actual driving, so their thinking process should also be taken into consideration. Hence, a choice reaction time is selected in this research. A range of different types of information are shown to a driver randomly, and the driver must respond differently to each different type of stimulus signal. The method for the driver’s reaction time test can be described as follows: The computer announces a number (1, 2 or 3) randomly, and the driver needs to press the right button as fast as possible. The period between announcing the number to pressing the button is defined as the driver’s reaction time. The flow chart of the reaction time test is shown in Figure 1.

The reaction time test has been shown to be effective when the driver’s hands were in the correct position. The reaction time value often increased if the driver was holding the steering wheel with only one hand, which may not exactly reflect a state of fatigue. Hence, test data captured under incorrect conditions should be eliminated. Additionally, swerving, changing lanes, overtaking and talking also led to abnormal data, so this data was also removed from the data analysis.

#### 2.1.2. Subjects

All subjects who held a driving license were screened for eligibility. Twelve males (60%) and eight females (40%) were voluntarily recruited for this research from the general public. Their ages ranged from 24 to 51, and their driving experience ranged from three years to 25 years. Before the experiments, the participants were given information on the research and they all consented to participate in the experiments. To ensure the effectiveness of the research, it was verified that none of the participants had medical contraindications such as disease, alcoholism, drug abuse or psychological or intellectual problems. It was also ensured that they had sufficient sleep, with no alcohol or coffee for 24 h prior to participation in the experiments.

#### 2.1.3. Experimental Procedure

A freeway scene from the simulator was chosen as the test scene. The speeds ranged from 80 to 120 km/h. The average driving time was approximately four to six hours. Before the experiment, all experimental devices were explained to each driver, including the biopac system and the reaction time test system. The driver was instructed to start driving when ready. The tester then started the biopac system and recorded the time. Physiological data was collected during the whole process. During the experiment, the tester recorded the driver’s reaction time reflecting the driver’s state every three min and recorded the data and the time. A new self-assessment method was proposed based on the Stanford Sleepiness Scale in order to allow the driver to also perform a self-assessment quickly and intimately (Table 1). Level 1 represents when the driver is in a state of vigilance. Level 2 represents when the driver has slight mental fatigue. Level 3 represents when the driver has serious mental fatigue. The self-assessment scale was recorded after each reaction time test to perform mutual verification of the classification results. The maximum driving time was six hours. If the subject became fatigued after four hours’ driving, the driving would be stopped and the driving duration would be four hours. If the subject did not become fatigued after four hours’ driving, the experiment would continue and stop only when the subject became seriously fatigued. The biopac systems were stopped and the finish time was recorded when the experiment was stopped.

### 2.2. Signal Processing

The EEG signal was divided into 60-second time epochs. The power spectral density (PSD) of α (8–13 Hz), β (13–30 Hz), δ (1–4 Hz) waves and the overall EEG (1–30 Hz) waves were extracted for each epoch using the fast Fourier transform and the Welch PSD. The energy of each wave was then calculated based on the integral of the PSD. The PSD of the α, β, δ and EEG waves (represented by α-PSD, β-PSD, δ-PSD and EEG-PSD) were chosen as the factors impacting the reaction time. The average power spectrum of the α, β and θ waves could be directly extracted from the biopac system. A Haar wavelet transform was used to eliminate noise, which can intuitively show changes in the three waves. A single wave cannot adequately represent the driver’s state at a point in time, due to other types of interference. The alpha activity and theta activity were mutually integrated to produce a more promising effect than alpha activity or theta activity alone [18]. In this research, three other ratio formulas, α/β, (α+θ)/β and α-PSD/β-PSD were also chosen as factors that impact the reaction time.

The ECG signal reflects the state of motion of the heart, which is controlled simultaneously by the sympathetic and parasympathetic nervous system. The heart rate and the heart rate variability (HRV) calculated from the ECG signal are regarded as the indicators that can be used to assess mental state. One of the statistical indicators of HRV is the standard deviation (SD) of the RR interval (where the RR interval is the time between consecutive R peaks in the ECG waveform, represented by $RR_{SD}$). $RR_{SD}$ can be calculated using the following Formula (1):

(1) $$RR_{SD}=\sqrt{\frac{\sum_{i=1}^{N}{\left(RR_{i}-\bar{RR}\right)}^{2}}{N}}$$

The psychological burden of the driver increases when they feel tired, which causes a change in heart rate and $RR_{SD}$. Hence, the heart rate and $RR_{SD}$ were also chosen as impacting factors of the reaction time.

### 2.3. Parameter Selection Based on Grey Correlation Analysis

Not all of the physiological impacting factors have a significant relationship to the reaction time. There are definite and indefinite conditions that can affect a driver’s mental state. Since this system is a gray process related to time, there is a potential relationship between the impacting factors and the reaction time. All of this data should be normalized using the following Formula (2):

(2) $$y_{i}=\frac{x_{i}-\underset{1\leq j\leq n}{\min}\left\{x_{j}\right\}}{\underset{1\leq j\leq n}{\max}\left\{x_{j}\right\}-\underset{1\leq j\leq n}{\min}\left\{x_{j}\right\}}$$

The calculation steps for the grey correlation analysis are shown as follows. First, the following parameters are established: the original matrix $x_{i}=(x_{i}(1),x_{i}(2),...,x_{i}(k),...)$ of the reaction time, α-PSD, β-PSD, δ-PSD, EEG-PSD, α-PSD/β-PSD, (α+θ)/β, α/β, the heart rate and $RR_{SD}$. $x_{i}(k)$ represents the original data value for factor *i* at time *k*, in minutes. The initialization transformed matrix $x_{i}^{\prime }(k)=x_{i}(k)/x_{i}(1)$ is evaluated and the difference sequence $\Delta_{0i}(k)$ is then evaluated using the following Formula (3):

(3) $$\Delta_{0i}(k)=\left|x_{0}^{i}(k)-x_{0}^{\prime }(k)\right|,\Delta_{0i}(k)=(\Delta_{0i}(1),\Delta_{0i}(2),...,\Delta_{0i}(k),...)$$

Finally, the correlation coefficient $\xi_{0i}(k)$ and the grey correlation degree $\gamma_{0i}$ are calculated using the Formulas (4) and (5):

(4) $$\xi_{0i}(k)=\frac{\underset{i}{\min}\;\underset{k}{\min}\;\Delta_{0i}(k)+\varphi \;\underset{i}{\max}\;\underset{k}{\max}\;\Delta_{0i}(k)}{\Delta_{0i}(k)+\varphi \;\underset{i}{\max}\;\underset{k}{\max}\;\Delta_{0i}(k)}$$

A resolution ratio (φ=0.5) is used to improve the significance of the difference between the correlation coefficients.

(5) $$\gamma_{0i}=\frac{1}{n-1}\sum_{k=1}^{n}\xi_{0i}(k)$$

### 2.4. Classification of Mental State Based on SVM

The parameter with the maximum degree of correlation with the reaction time was used as the input variable of the classification model. Successive physiological data collected from experiments was used to reflect each driver’s state. However, there is a limited quantity of factors that can be used for the reaction time, since collection of too many factors may affect driving safety, while too few factors may not be representative. Hence, the quantity of factors used to determine the reaction time was much less than the amount of physiological data. SVM can accurately solve the classification problem by using only a few training samples rather than a large number of training samples, which is suitable for this research.

There are two types of sample sets that are linearly separable $\left(x_{i},y_{i}\right),i=1,\cdots ,n,x_{i}\in R^{d}$, $y_{i}\in \left\{-1,+1\right\}$. The linear discriminant function is f(x)=ω⋅x+b. The corresponding classification equation is ω⋅x+b=0. The linear discriminant function is normalized, which ensures that both types of samples meet |f(x)|≥1. The samples with the minimum distance to the classification hyperplane should also meet f(x)=1. If the sample can be assigned to the correct category, the condition should meet Formula (6):

(6) $$y_{i}\left[\left(\omega \cdot x_{i}\right)+b\right]-1\geq 0,i=1,\cdots ,n$$

The interval for classification is 2/‖ω‖. The maximum interval is equivalent to the minimum value of ${\left\|\omega \right\|}^{2}$. Hence, the classification hyperplane that meets Formula (6) and has the minimum $\frac{1}{2}{\left\|\omega \right\|}^{2}$ value is the optimal classification hyperplane. The minimum of $\phi \left(\omega \right)=\frac{1}{2}{\left\|\omega \right\|}^{2}$ can be evaluated under the conditions of Formula (6). The Lagrange function is defined to solve the problem above using the Formula (7):

(7) $$L\left(\omega ,b,\alpha \right)=\frac{1}{2}{\left\|\omega \right\|}^{2}-\sum_{i=1}^{n}\alpha_{i}\left[y_{i}\left(\omega \cdot x_{i}+b\right)-1\right]$$

where the Lagrange multiplier is $\alpha_{i}$ and $\alpha_{i}$ is equal to or greater than zero. ω, b and $\alpha_{i}$ are evaluated to obtain the minimum value of Formula (7) by using the Formula (8):

(8) $$\left\{ \begin{array}{l} \frac{\partial L}{\partial \omega }=0\Rightarrow \omega =\sum_{i=1}^{n}\alpha_{i}y_{i}x_{i}\\ \frac{\partial L}{\partial b}=0\Rightarrow \sum_{i=1}^{n}\alpha_{i}y_{i}=0\\ \frac{\partial L}{\partial \alpha_{i}}=0\Rightarrow \alpha_{i}\left[y_{i}\left(\omega \cdot x_{i}+b\right)-1\right]=0 \end{array}\right.$$

According to the conditions above, assessment of the optimal classification hyperplane can be transformed into a dual problem of convex quadratic programming optimization using the following Formula (9):

(9) $$\left\{ \begin{array}{l} \max\sum_{i=1}^{n}\alpha_{i}-\frac{1}{2}\sum_{i=1}^{n}\sum_{j=1}^{n}\alpha_{i}\alpha_{j}y_{i}y_{j}\left(x_{i}\cdot x_{j}\right)\\ s.t.i\geq 0,i=1,\cdots ,n\\ \sum_{i=1}^{n}\alpha_{i}y_{i}=0 \end{array}\right.$$

where the Lagrange multiplier is $\alpha_{i}$. There is a unique solution in the convex quadratic programming optimization. If $\alpha_{i}^{*}$ is the optimization, then $\omega^{*}$ is equal to $\sum_{i=1}^{n}\alpha_{i}^{*}y_{i}x_{i}$. The support vector contains the samples that satisfy the condition that $\alpha_{i}^{*}$ is not equal to zero are the support vector. Hence, the weight coefficient vector of the optimal classification hyperplane is the linear combination of the support vector. $b^{*}$ is the classification threshold that can be evaluated from the constraint condition $\alpha_{i}\left[y_{i}\left(\omega \cdot x_{i}+b\right)-1\right]=0$.

The optimal classification function can be obtained by solving the problems above using the following Formula (10).

(10) $$f\left(\omega \right)=\mathrm{sgn}\left\{\left(\omega \cdot x\right)+b\right\}=\mathrm{sgn}\left\{\sum_{i=1}^{n}\alpha_{i}^{*}y_{i}\left(x_{i}\cdot x\right)+b^{*}\right\}$$

Under some circumstances, the optimal classification hyperplane cannot be completely separated into two types of samples. The misclassified samples can be allowed by introducing the relaxation factor ξ to the Formula (6) basis, which makes the samples approximately linearly separable. At this time, the classification hyperplane ω⋅x+b=0 should satisfy the condition of the Formula (11):

(11) $$y_{i}\left[\left(\omega \cdot x_{i}\right)+b\right]\geq 1-\xi_{i},i=1,\cdots ,n$$

Each sample $x_{i}$ is classified correctly when $\xi_{i}$ is greater than zero and less than one. If $\xi_{i}$ is equal to or greater than one, sample $x_{i}$ is incorrectly classified. A penalty term $C\sum_{i=1}^{n}\xi_{i}$ is added to meet $\phi \left(\omega ,\xi \right)=\frac{1}{2}{\left\|\omega \right\|}^{2}+C\sum_{i=1}^{n}\xi_{i}$, where C is the penalty factor that is a positive constant. Hence, Formula (9) can be optimized to obtain Formula (12):

(12) $$\left\{ \begin{array}{l} \max\sum_{i=1}^{n}\alpha_{i}-\frac{1}{2}\sum_{i=1}^{n}\sum_{j=1}^{n}\alpha_{i}\alpha_{j}y_{i}y_{j}\left(x_{i}\cdot x_{j}\right)\\ s.t.0\leq \alpha_{i}\leq C,i=1,\cdots ,n\\ \sum_{i=1}^{n}\alpha_{i}y_{i}=0 \end{array}\right.$$

The original space can be mapped to a high dimensional space using a nonlinear transformation, in order to solve the problem of nonlinear classification. A radial basis function (RBF) kernel function is used to obtain the optimal classification hyperplane in this transformation. The RBF kernel function is $\exp\left\{-\frac{{\left\|x-x_{i}\right\|}^{2}}{\sigma^{2}}\right\}$. $\frac{1}{\sigma^{2}}$ is the radius of the kernel function. It is greater than zero and can be represented by g. Hence, the objective function is as shown in Formula (13):

(13) $$Q\left(\alpha \right)=\sum_{i=1}^{n}\alpha_{i}-\frac{1}{2}\sum_{i,j=1}^{n}\alpha_{i}\alpha_{j}y_{i}y_{j}K\left(x_{i},x_{j}\right)$$

The corresponding classification function is as shown in Formula (14):

(14) $$f\left(x\right)=\mathrm{sgn}\left(\sum_{i=1}^{n}\alpha_{i}^{*}y_{i}K\left(x_{i},x_{j}\right)+b^{*}\right)$$

**Figure 2:**
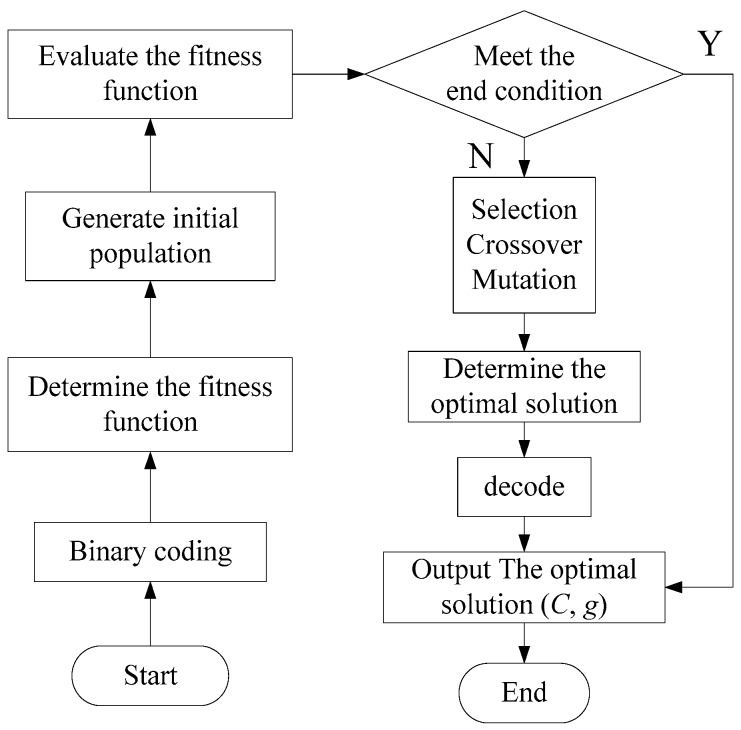
The flow chart of the Genetic Algorithm (GA).

The penalty factor C represents the error tolerance. The higher the value of C, the more likely the error cannot be tolerated. An exorbitant value of C may decrease the generalization ability of the classifier. Hence, the C and g values have a significant effect on the classification accuracy. The Genetic Algorithm (GA), proposed by Professor Holland and his students, is used to search for the minimum value of C and the corresponding value of g [19]. The flow chart for the GA is shown in Figure 2.

1. Population initialization. Encode the individuals to binary code. The code symbol set consists of 0 and 1, which comprises a binary symbol set {0,1}. Each individual genotype in the binary symbol set is a binary encoding symbol string.
2. Determine the fitness function. The accuracy of the Cross Validation (CV) is regarded as the fitness function of the GA model. Cross Validation is a statistical method that can verify the performance of the classifier. Familiar CV methods include the Hold-Out Method, the K-fold Cross Validation (K-CV) and the Leave-One-Out Cross Validation (LOO-CV). In this research, K-CV was chosen for the fitness function. The original data is divided into three groups with approximately equal sizes. One subset is a validation set, and the other two subsets are the training sets. Three values of the classification accuracy are then obtained from the three validation sets of the models. The average accuracy is regarded as the performance index of the classifier in K-CV.
3. Selection operation. Roulette Selection, based on the strategy of fitness proportion selection, was chosen as the method for the selection operation. If the selective probability of each individual i is $p_{i}$, then the calculation method is shown as Formulas (15) and (16):
  (15) $$f_{i}=k/F_{i}$$
  (16) $$p_{i}=\frac{f_{i}}{\sum_{j=1}^{N}f_{j}}$$
  where $F_{i}$ is the fitness value of each individual i, k is a coefficient and N is the size of the population.
4. Crossover selection. The method of crossover selection, where chromosome $a_{k}$ crosses chromosome $a_{l}$ at position j, is shown in Formula (17):
  (17) $$\begin{array}{l} a_{kj}=a_{kj}\left(1-b\right)+a_{lj}b\\ a_{\;lj}=a_{\;lj}\left(1-b\right)+a_{kj}b \end{array}\}$$
  where b is a random number in the range [0,1].
5. Mutation operation. Choose the jth gene $a_{ij}$ of the ith individual and vary this gene using the following Formula (18):
  (18) $$a_{ij}=\left\{ \begin{array}{l} a_{ij}+\left(a_{ij}-a_{\max}\right)\times f\left(g\right),r>0.5\\ a_{ij}+\left(a_{\min}-a_{ij}\right)\times f\left(g\right),r\leq 0.5 \end{array}\right.$$
  where $a_{\max}$ is the upper bound of gene $a_{ij}$, $a_{\min}$ is the lower bound of gene $a_{ij}$, $f\left(g\right)=r_{2}{\left(1-g/G_{\max}\right)}^{2}$, $r_{2}$ is a random number, g is the current iteration, $G_{\max}$ is the maximum number of evolutions and r is a random number in the range [0,1].

## 3. Results and Discussion

### 3.1. Results of the Grey Correlation

Twenty groups of datasets were analyzed. The results of the grey correlation analysis are shown in Table 2. From the results of the grey correlation analysis comparing the seven impacting factors that were obtained from the EEG signal, it was found that the EEG-PSD has the greatest degree of correlation (average 0.9783) with the reaction time. The comparison of the two impacting factors that were obtained from the ECG signal found that $RR_{SD}$ has the greatest degree of correlation (average 0.8580) with the reaction time. The degree of correlation of the heart rate (average 0.8427) is approximately equal to the degree of correlation of $RR_{SD}$. It can be concluded that the EEG signal has a larger correlation with the reaction time than the ECG signal. This phenomenon is in accordance with the objective fact that the reaction time of the driver is controlled by the brain, and the motion of the heart shows less of a direct effect on the reaction time. However, the EEG-PSD contains all of the frequency components of the three waves (1–30 Hz), which does not accurately represent the state of fatigue. It can be seen that α/β has the greatest correlation with the reaction time (average 0.9386) out of the three types of mutual integration factors that were extracted and calculated from the EEG signal. Additionally, β-PSD has the greatest correlation with the reaction time (average 0.8756) out of the α, β and δ waves. It is known that β waves appear when the brain is excited or alert, which indicates that an alert state contributes to an improvement in a driver’s reaction ability.

### 3.2. The Results of SVM Model Classification

The reaction time and the values of α/β were chosen as the input variables based on the grey correlation analysis. There were 242 dataset groups selected from the above experiments for verification. A combined two-class classifier was used to divide the mental state into three levels. Levels 1 and 2 were considered to be the same category (represented by Category I), while Level 3 was considered to be a different category (represented by Category II). The two-class classifier first divided the datasets into Category I or Category II (Level 3), and then the classifier divided Category I into Level 1 and Level 2. From the 242 groups of datasets, 142 groups of datasets were used as the training datasets, and 100 groups of datasets were used as the testing datasets. There were 200 iterations, the population quantity was 20, and the parameter for the cross validation was 10. The best value of C was found to be 0.4031 and the best value of g was found to be 7.6761 after GA optimization. The classification accuracy in K-CV is 90.1408%. The results of the SVM classification model are shown in Table 3. In the first classification, for Level 3, 21 datasets were correctly classified out of a total of 24 datasets, resulting in a sensitivity of 87.5%. For Level 1 and Level 2, 65 datasets were correctly classified out of a total of 76 datasets, resulting in a specificity of 85.53%. This resulted in an accuracy of 86%. In the second classification, for Level 2, 25 datasets were correctly classified out of a total of 30 datasets, resulting in a sensitivity of 83.33%. For Level 1, 40 datasets were correctly classified out of a total of 46 datasets, resulting in a specificity of 86.96%. This resulted in an accuracy of 85.53%.

A confusion matrix contains information about actual and predicted classifications done by a classifier. Performance of such a classifier can be evaluated using the data in the matrix. Table 4 shows the confusion matrix of mental state classification. More results can be obtained from the confusion matrix to evaluate the SVM classification model. For the first classification, the false positive rate (FP) is 1.32%, the false negative rate (FN) is 12.5%, the precision (P) is 95.45%. For the second classification, the FP is 13.04%, the FN is 13.33%, the P is 80.65%.

Under the constraint that the reaction time must be chosen as one of the input variables, the accuracy of SVM after changing the other input variables should also be analyzed. The first classification was analyzed after changing the input variables. The results are shown in Table 5.

EEG signal is sensitive to variations in reaction time. It is known that α waves occur when a driver is relaxed, or when attention levels are decreased. In particular, α waves are more obvious than other waves during monotonous driving tasks [20]. However, θ waves primarily occur when a driver is in a sleepy state or there is an increased task demand. Hence, mutual integration of the α waves and the β waves has shown more promising results than individual detection of α waves, θ waves or β waves alone. Moreover, since periods in both Level 1 and Level 2 were suitable for driving, while periods in Level 3 were not, there is no easily distinguishable boundary between Level 1 and Level 2. However, the accuracy of SVM is 86%, which proves the applicability of SVM for mental state classification. It can be concluded that the accuracy will decrease if a physiological parameter with a lower correlation to the reaction time is chosen as the input variable of the SVM model. The accuracy also decreases if the number of input variables increases. Hence, physiological parameter optimization based on grey correlation analysis has a significant influence on the accuracy of the SVM classification.

**Figure 3:**
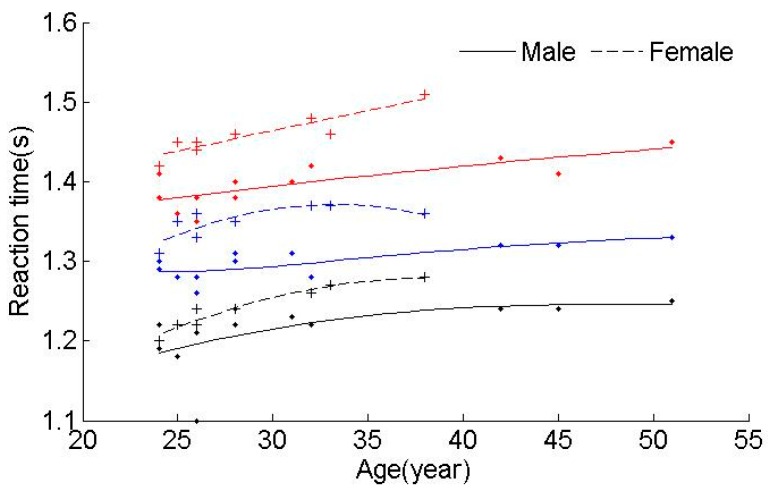
The relationship between reaction time, age, gender and mental fatigue levels.

Driving fatigue is influenced by many types of factors. Driving over a long period of time eventually results in mental fatigue or drowsiness, although mental fatigue or drowsiness may also occur during shorter drives. Hence, it is hard to establish a formula that can be used to define the relationship between the reaction time and α/β. Fatigue classification can accurately solve this problem and eliminate the time factor. The relationship between the reaction time and α/β can then be obtained from the results of the classification. Due to different individual features, the results are also different. Figure 3 shows that the reaction time changes with age and gender for different mental levels. In Figure 3, the black data points represent Level 1, the blue data points represent Level 2, and the red data points represent Level 3. Table 6 gives the results that show that the reaction time changes with mental levels.

It is obvious that Level 3 has the largest average reaction time, while Level 1 has the lowest average time, which indicates that the reaction time becomes longer as mental fatigue accumulates. It can be concluded that females have a quicker increase in reaction time than males as driving fatigue accumulates. The stamina of females is poorer than males, which indicates that females may get fatigued faster than males. Moreover, there is a difference between males and females for the same mental level. The reaction time of females has been found to be longer than males for each mental level. The higher the level, the bigger the gap between males and females.

Age is another significant factor that can influence the reaction time. The results of the average reaction time of different age groups are shown in Table 7. The subjects were divided into two groups: age 20–30 years and age over 30 years. Proficiency of driving is another important factor that has an impact on the reaction time. A young driver, who is not experienced at driving, will be over-anxious, and can easily concentrate on individual points. This type of driver may have a long reaction time. In contrast, an older driver with rich experience in driving, may be able to provide quick responses to an emergency. This type of driver can compensate for deficiencies of old age.

### 3.3. The Analysis of the Reaction Time in the Time Domain

**Figure 4:**
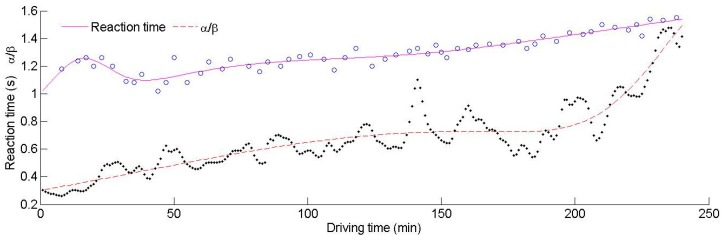
The reaction time and α/β as time increases.

The relationship between the reaction time, α/β and driving fatigue needs be analyzed in relation to the time domain. A representative experiment during which the driver progressed through a process of being awake to asleep is shown in Figure 4. The aim of this experiment was to observe the consistency of the tendency of the two parameters to show particular patterns for different mental states, especially during fatigue. The reaction time can reflect the reliability of the driver. As the length of time driving increased, the driver became fatigued, which manifested as increased distraction levels and longer reaction times. There was an increase in α waves and a decrease in β waves when the driver felt tired. Hence, the value of α/β was seen to increase as fatigue accumulated.

It can be concluded from Figure 4 that α/β increases with time, and the reaction time decreases first and then increases. This indicates that the reaction time does not always have a positive correlation with α/β, especially during the initial period. To analyze the relationship between α/β, the reaction time and driving fatigue, the driving process has been divided into three periods.

The first period (0–44 min): The driver had just started to drive and was unfamiliar with the driving environment during this period. However, the driver was alert during this period, so α/β had low values. Due to unfamiliarity with the environment, there was a high workload on the driver. During the transition from increased mental workload to mental fatigue, there was an evident increase in α waves and a decrease in β waves, resulting in a quick growth of α/β. As the driver adapted to their environment, the rate of growth of α/β reduced, indicating that the driving performance had become stable. The reaction time first increased, then decreased and finally increased again during this period. The degree of familiarity depended on the driver’s “internal” state. Poorer driver performance may have been due to an increase in the quantity of demanding tasks. The situational awareness of the driver also started to reduce. Hence, the driver was in an unstable state at the beginning, leading to a remarkable fluctuation in the reaction time. The reaction time shortened after 23 min, indicating that the driver had become familiar with the driving task and the reaction time test. Hence, the measured reaction time after 23 min was effective. The reaction time was at its lowest value at the 44th minute, before increasing gradually. Moreover, the volatility of the reaction time was large (0.0068) prior to 44 min, which also indicates that the driver’s behavior was in an unstable state. However, the volatility decreased after 44 min, indicating that the driver’s behavior had become stable and reliability was starting to increase.

The second period (45–188 min): Actually, there was no obvious boundary between the first period and the second period. Due to the instability of the driver at the start of driving, an abnormal reaction time was observed. Hence, the first period was defined to justify the large fluctuations in reaction time. In fact, both the first period and the second period are suitable for driving. During the second period, the growth rate of α/β slowed down, and α/β maintained a relatively stable value. At the beginning of the driving task, the driver was required to remember lots of information relating to their environment. After a certain length of time driving, the driver was able to allocate some attention to secondary tasks. During this period, the reaction time tended to increase quickly at the beginning and then continued to increase at a slower pace. The volatility of the reaction time (0.0065) was smaller than before (0.0068), indicating that the driver was in a stable state. The reaction time reached a maximum after 117 min. The driver started to feel some fatigue as the driving time accumulated, which resulted in a slowdown in the increase in reaction time. Additionally, the reaction time was influenced by many other factors such as swerving and overtaking that may have contributed to longer reaction times. For example, the reaction time at the 50th minute had a large difference with the adjacent reaction times before and after. On further investigation, it can be seen that the driver was overtaking during this period, and therefore, for the sake of driving safety, the driver could not press the button immediately. Hence, the measured values do not always reflect the driver’s mental state precisely. During this period, the driver has adapted to the environment, and his reliability was the highest, which was the best state for driving. However, there were strong fluctuations in α/β later on in this period. The driver began to feel slight fatigue which he attempted to resist, but he struggled to keep alert. The reaction time had small fluctuations during this time.

The third period (189–240 min): The driver became fatigued quickly as the monotonous driving task progressed further. As the driving time accumulated, the driver started to feel sleepier and lost interest in remaining awake. The slope of α/β increased quickly after 188 min, and the value of α/β rose with astonishing speed. The reaction time also increased more quickly than before. The driver was fatigued and the reliability of the driver was low during this period, leading to a decrease in control of the vehicle. It can be concluded that a fatigued state can reduce a driver’s ability to respond to stimuli. It is not appropriate to continue driving any further during this period.

**Figure 5:**
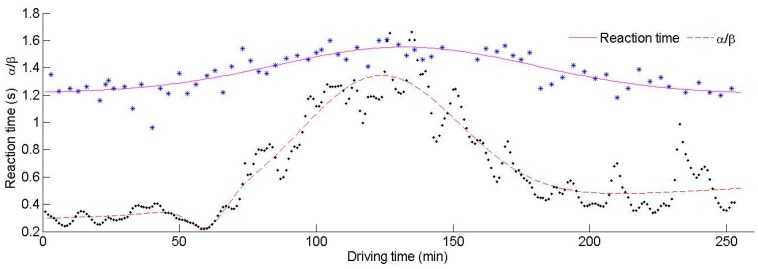
The reaction time and α/β as time increases.

However, it is important to stress that although the brain activity can be described as a series of transitions from an “alert state” to a “fatigued state” and from there to a “drowsy state” the transitions do not necessarily occur in this order [20]. Figure 5 shows another experiment where the driver experienced a process of transitioning from alert to fatigued and then to alert again.

The driver was vigilant during the initial period of the experiment. Hence, the value of α/β stayed low and remained relatively stable. The reaction time was a little longer at the start of driving, which was quite similar to the first experiment and can be attributed to the driver needing time to adapt to their driving environment. The reaction time had an obvious fluctuation between 33 min and 46 min. This was due to the driver being in a complex traffic environment, and performing tasks such as overtaking or swerving. The value of α/β increased rapidly after 50 min. The reaction time had a large fluctuation again. The driver felt sleepy after 90 min and was frequently yawning. The difference between this experiment and the first experiment was that the driver became fatigued after only one hour of driving. This only occurred after three hours in the first experiment. The reason for the quicker fatigue was due to physiological cycles. The experiment began at 10:30 a.m. and after 90 min reached afternoon time. The driver became drowsy after 150 min driving and a collision occurred. At the time of the collision, the driver was inattentive and unconscious, and had a long reaction time. After the collision, the driver sobered and the brain struggled to keep alert. Hence, β waves occurred during these conditions of vigilance, which had an increased attention level, and the value of α/β and the reaction time began to decrease. The driving fatigue started to accumulate again after 210 min of driving, resulting in an increase in α/β. However, the reaction time did not show an obvious decrease due to self-regulation by the driver.

## 4. Conclusions

In this paper, physiological signals and the reaction time of drivers were collected from twenty experiments. Nine types of physiological factors impacting the reaction time were obtained in order to analyze their degree of correlation with the reaction time based on grey correlation analysis. The results of the grey correlation analysis were used to select the input variable of the classification model. SVM was used to divide the mental state into three levels and GA was used to optimize the penalty factor.

The results have shown that α/β has the largest correlation with the reaction time. The classification results show an accuracy of 86%, a sensitivity of 87.5% and a specificity of 85.53%, which proves the applicability of SVM for mental state classification. It can be concluded that the classification accuracy decreases if an input variable with a lower correlation with the reaction time is chosen. Hence, physiological parameter optimization based on grey correlation analysis can contribute to improving the classification accuracy. The reaction time shows large fluctuations during initial driving and increases as fatigue accumulates after the driver has adapted to their driving environment. The reaction time has a positive correlation with α/β when the driver’s behavior becomes stable. Females have been shown to have a poorer reaction ability than males as driving fatigue accumulates. The higher the mental state, the bigger the gap between males and females. Elderly drivers also have longer reaction times than the young, although rich driving experience may compensate for age-related deficiencies. If the testing reaction time is 16.72% higher than the average value during an alert, it is not inappropriate for driving, which can eliminate individual factors.

In contrast with previous research, this paper has determined the physiological factor with the largest correlation to the reaction time, which can optimize the SVM classification model and promote its accuracy. Previous research has studied the variation of reaction time with age, gender and mental workload, while this research has analyzed the reaction ability of a driver by combining physiological factors when a driver transitions from being awake to sleepy. The results also show a proportional change in reaction time for different ages and genders during fatigue. In particular, online detection of reaction times related to fatigue or drowsiness can be performed using only a simple device, which is safer and more comfortable for users. Future research should focus on human–machine interaction of the reaction time test system and conducting road experiments.

## Figures and Tables

**Table 1:** Self-assessment scale.

Mental State	Scale Statement
Level 1	Alert, Able to Concentrate
Level 2	Responsive but Not Fully Alert, Not at Peak
Level 3	Losing Interest in Remaining Awake, Somewhat Foggy, Sleepy

**Table 2:** The degree of correlation between the physiological parameters and the reaction time.

Group	α-PSD	β-PSD	δ-PSD	EEG-PSD	α-PSD/β-PSD	(α + θ)/β	α/β	Heart Rate	$RR_{SD}$
1	0.7556	0.6865	0.6798	0.9865	0.8047	0.6965	0.8440	0.8208	0.8431
2	0.6301	0.9238	0.8032	0.9713	0.7800	0.7012	0.9637	0.7950	0.7651
3	0.7501	0.9590	0.8895	0.9835	0.8038	0.8184	0.9656	0.8019	0.7677
4	0.7900	0.7812	0.6999	0.9706	0.8496	0.7422	0.8958	0.7996	0.8196
5	0.8386	0.9755	0.9641	0.9935	0.8980	0.9315	0.9870	0.9184	0.9104
6	0.7626	0.8169	0.7660	0.9882	0.8306	0.8208	0.9190	0.7979	0.8637
7	0.9199	0.9116	0.8635	0.9918	0.9238	0.9063	0.9665	0.9411	0.9341
8	0.8068	0.8957	0.8013	0.9149	0.8679	0.7673	0.9822	0.7518	0.8288
9	0.9079	0.9395	0.9345	0.9934	0.9261	0.9253	0.9749	0.9462	0.9438
10	0.7167	0.7954	0.7661	0.9787	0.7894	0.6806	0.9242	0.8550	0.8249
11	0.8341	0.9612	0.9162	0.9885	0.9251	0.8457	0.9678	0.9106	0.9366
12	0.8601	0.9684	0.9610	0.9948	0.9340	0.9021	0.9883	0.9276	0.9252
13	0.8728	0.8566	0.8175	0.9840	0.8051	0.7999	0.9167	0.7529	0.8325
14	0.8961	0.8125	0.7630	0.9783	0.7652	0.7862	0.8900	0.7941	0.7912
15	0.8870	0.9125	0.8532	0.9895	0.9151	0.8767	0.9683	0.8992	0.8924
16	0.8311	0.9687	0.9663	0.9949	0.9193	0.8833	0.9958	0.9264	0.9126
17	0.8267	0.8574	0.8132	0.9846	0.7296	0.8031	0.8759	0.6805	0.7808
18	0.8022	0.7612	0.7093	0.9009	0.8630	0.7178	0.9218	0.7680	0.8510
19	0.9005	0.8997	0.8566	0.9863	0.9227	0.8890	0.9706	0.9194	0.9082
20	0.7967	0.8279	0.7658	0.9919	0.8588	0.8086	0.8548	0.8471	0.8275
Average	0.8193	0.8756	0.8295	0.9783	0.8556	0.8151	0.9386	0.8427	0.8580

PSD = Power spectral density; EEG = Electroencephalograph; RR: RR interval is the time between consecutive R peaks in the ECG waveform; SD = Standard deviation.

**Table 3:** The results of support vector machine (SVM) classification.

The First Classification		The Second Classification	
Correct Classification of Level 3	21	Correct Classification of Level 2	25
Correct Classification of Level 2 and Level 1	65	Correct Classification of Level 1	40
the Total of Level 3	24	The Total of Level 2	30
the Total of Level 2 and Level 1	76	The Total of Level 1	46
Sensitivity	87.5%	Sensitivity	83.33%
Specificity	85.53%	Specificity	86.96%
Accuracy	86%	Accuracy	85.53%

**Table 4:** The confusion matrix of mental state classification.

Mental State		Predicted Class		
		Level 1	Level 2	Level 3
Actual Class	Level 1	40	6	0
	Level 2	4	25	1
	Level 3	0	3	21

**Table 5:** Analysis of changing input variables.

Input Variables	Accuracy
The Reaction Time, α/β	86%
The Reaction Time, RR Interval	42%
The Reaction Time, α/β, RR Interval	81%
The Reaction Time, α/β, Heart Rate	49%

**Table 6:** The average reaction time of different mental levels.

Gender	Level 1 (s)	Level 2 (s)	Level 3 (s)	Growth Rate from Level 1 to Level 2	Growth Rate from Level 1 to Level 3
Male	1.21	1.30	1.40	7.44%	15.70%
Female	1.24	1.35	1.46	8.87%	17.74%
Growth Rate	2.44%	3.98%	4.39%	Average 8.16%	Average 16.72%

**Table 7:** The average reaction time of different age groups.

Age Groups (Years Old)	Level 1 (s)	Level 2 (s)	Level 3 (s)
20–30	1.21	1.31	1.41
Over 30	1.25	1.33	1.45
